# The Distribution of Cervical Human Papillomavirus Infection Among Chinese Adult Women

**DOI:** 10.1089/whr.2023.0035

**Published:** 2023-09-29

**Authors:** Xiaojing Ma, Xianglei Kong

**Affiliations:** ^1^ The Center of Health Examination, Shandong Provincial Qianfoshan Hospital, The First Affiliated Hospital of Shandong First Medical University, Jinan, P.R. China.; ^2^ Department of Nephrology, Shandong Provincial Qianfoshan Hospital, The First Affiliated Hospital of Shandong First Medical University, Jinan, P.R. China.

**Keywords:** cervical cancer, epidemiology, human papillomavirus, subtypes

## Abstract

**Background:**

The characteristics and genotypic distribution of human papillomavirus (HPV) infection differ in different countries and regions. The purpose of this study was to analyze the distribution of 27 HPV subtypes among adult women receiving health examinations in Jinan, China.

**Methods:**

A retrospective analysis was performed to analyze HPV subtype infection among adult women who underwent cervical cancer screening from January 1, 2020, to December 31, 2020.

**Results:**

Among 4746 women receiving HPV testing, 682 cases were positive, representing 14.4% of patients. In total, 514 cases were infected by a single HPV subtype (75.4%), 115 cases had dual infections (16.9%), and 53 cases had multiple infections (7.7%). Among the 682 cases of HPV infection, 503 cases (73.8%) were high-risk HPV infections. The most common high-risk HPV strains were HPV 52 (23.3%), HPV 16 (13.5%), and HPV 58 (12.7%). Low-risk HPV strains infected 179 cases (26.2%). The most common genotypes were HPV 61 (25.7%), HPV 81 (21.2%), and HPV 55 (15.6%).

**Conclusion:**

The HPV infection rate in healthy women was high, and the mixed infection rate was as high as 24.6%, highlighting the need for increased attention to this virus.

## Background

The incidence of cervical cancer ranks fourth in the world among female-related cancers, second only to breast cancer, colorectal cancer, and lung cancer.^1^ According to the latest global cancer burden data released by the International Agency for Cancer Research of the World Health Organization in 2020, ∼110,000 new cases of cervical cancer and 60,000 deaths in China were reported in 2020.^2^ Therefore, the prevention and treatment of cervical cancer is particularly arduous. In China, the incidence and mortality of cervical cancer are significantly increasing among young women.^3^ At present, cervical cancer has become the second most common cause of cancer and the third leading cause of cancer death among young women in China (15–44 years old).^4^ Human papillomavirus (HPV) infection is considered to be the main cause of cervical cancer.^5^

Recent epidemiological studies have shown that the HPV infection rate may be as high as 80% among sexually active women.^6^ At present, there is no effective treatment for HPV infection. Prophylactic HPV vaccines are currently the best method to prevent and control cervical cancer. Three types of HPV vaccines are available on the market: bivalent vaccines (for HPV 16, 18), tetravalent vaccines (for HPV 16, 18, 6, 11), and nine-valent vaccines (for HPV 6, 11, 16, 18, 31, 33, 45, 52, 58). These vaccines do not completely cover all major types of HPV infection, and most HPV vaccines are developed based on epidemiological data from Western countries. In fact, the characteristics and genotypic distribution of HPV infection differ in different countries and regions.^7^

The purpose of this study was to investigate the epidemiological characteristics of HPV infection in healthy women in Jinan, China, and to provide a reference for cervical cancer screening strategies and the clinical application of HPV vaccines in Jinan.

## Methods

### Participants

This is a routine cervical cancer screening in the health management center of a tertiary hospital. Adult women who underwent physical examination in our hospital from January 2020 to December 2020 were selected as the study subjects. Patients with a previous history of cervical cancer, surgical history of cervical precancerous lesions, and previously confirmed HPV infection were excluded. If the results of two or more HPV tests within 1 year were available, only the latest result was counted. All the participants signed the informed consent form, and the study was approved by the Ethics Committee of Shandong Provincial Qianfoshan Hospital.

### HPV detection

All specimens were collected by gynecologists according to the standard operating procedure, and the cervical brush was inserted into the cervical tube and rotated clockwise for three to five turns. The cervical brush was removed and put into the preservation solution for examination. Polymerase chain reaction (PCR) technology was applied to assess HPV viral DNA following the product's instructions. The HPV DNA test was performed using a Luminex-based suspension beads array to identify HPV types. The experimental protocol includes DNA extraction, PCR amplification, bead-coated hybridization, and digital signal processing. This method detected 17 high-risk HPV strains (16, 18, 26, 31, 33, 35, 39, 45, 51, 52, 53, 56, 58, 59, 66, 68, 82) and 10 kinds of low-risk HPV strains (6, 11, 40, 42, 43, 44, 55, 61, 81, 83).

### Statistical analysis

The measurement data with a normal distribution are expressed as means ± standard deviation, and the measurement data that do not conform to a normal distribution are represented by the interquartile range. The counting data are expressed as a percentage. The prevalence rate of HPV infection was analyzed, and the difference in HPV infection rates in different age groups was analyzed. The prevalence rates of single infection and mixed infection were calculated. The data were analyzed using SPSS statistical software. The difference was statistically significant (*p* < 0.05). A significant difference was noted between the two groups (*p* < 0.05).

## Results

Among the 4746 patients who underwent HPV testing, 682 cases (14.4%) were HPV positive, and the average age was 43.2 ± 10.6 years (21–76 years). Among the 682 cases of HPV infection, 514 cases (75.4%) were single infections, and 115 cases (16.9%) were dual infections. The common types of dual infections were HPV (type 52 + 39) and HPV (type 53 + 81). Multiple infections were found in 53 cases (7.7%) (Table 1). Among the multiple infections, the common types were HPV 56, 59, 53 and HPV 51, 11, 55.

**Table 1.** Baseline Character of the Study Population (*n* = 682)

Character	***n*** (%)
Age (years)	43.2 ± 10.6
Age group (years), *n* (%)	
<30	72 (10.6)
30–39	243 (35.6)
40–49	186 (27.3)
50–59	135 (19.8)
≥60	46 (6.7)
Infection pattern, *n* (%)	
Single infection	514 (75.4)
Dual infection	115 (16.9)
Multiple infection (≥3)	53 (7.7)

**FIG. 1.**
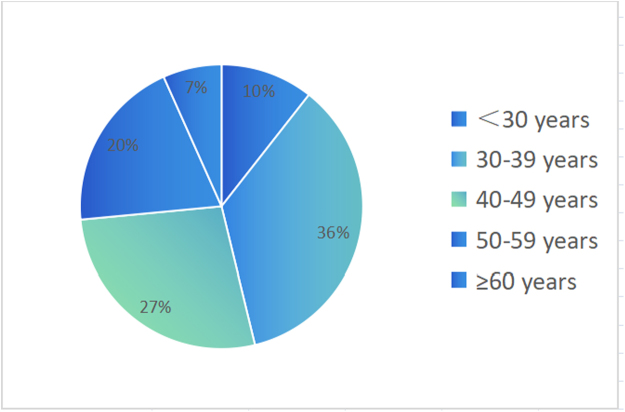
The proportion of HPV positive in different age groups (%). HPV, human papillomavirus.

Among these cases, the rates of HPV infections in each group were as follows: <30 years old, 72 cases (10.6%); 30–39 years old, 243 cases (35.6%); 40–49 years old, 186 cases (27.3%); 50–59 years old, 135 cases (19.8%); and ≥60 years old, 46 cases (6.7%). The infection rate was the lowest in the ≥60 years old group (Fig. 1).

HPV infection rates were assessed in different age groups. In the <30 years old group, there were 54 cases of single infection (75.0%), 14 cases of double infections (19.4%), and 4 cases of multiple infections (5.6%). In the group of patients between 30 and 39 years old, there were 177 cases of single infection (72.8%), 48 cases of double infections (19.8%), and 18 cases of multiple infections (7.4%). In the group of patients between 40 and 49 years old, there were 155 cases of single infection (83.3%), 18 cases of dual infections (9.7%), and 13 cases of multiple infections (7.0%).

**FIG. 2.**
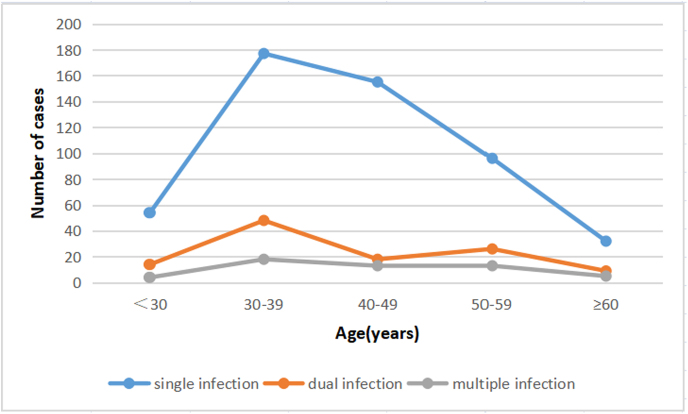
The distribution of cases of HPV infection in different age groups.

In the group of patients between 50 and 59 years old, there were 96 cases of single infection (71.1%), 26 cases of dual infections (19.3%), and 13 cases of multiple infections (9.6%). In the group of patients older than 60 years, there were 32 cases of single infection (69.6%), 9 cases of dual infections (19.6%), and 5 cases of multiple infections (10.9%) (Fig. 2).

### Distribution of different types of HPV infection

**FIG. 3.**
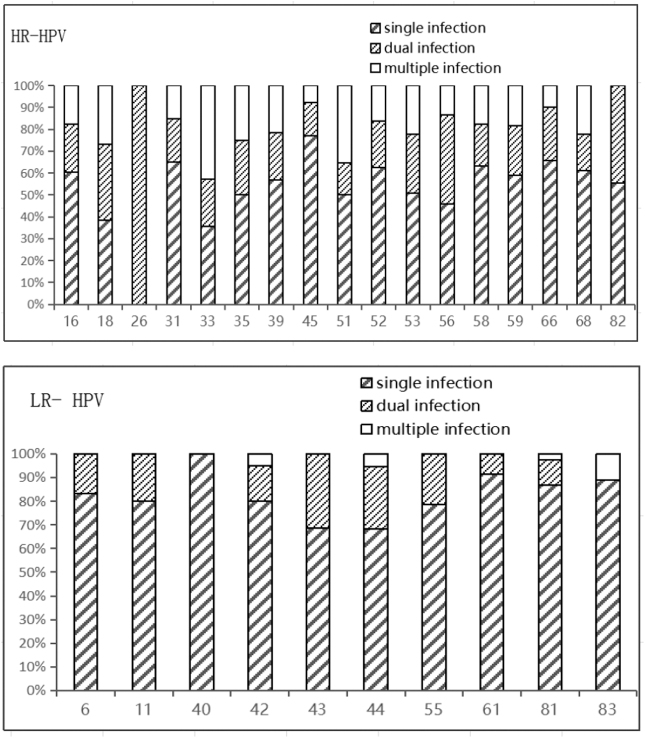
Distribution of different types of HPV infection. HR-HPV, high-risk human papillomavirus strains; LR-HPV, low-risk human papillomavirus strains.

Among the 682 cases of HPV infection, high-risk HPV infection accounted for 503 cases (73.8%), and the most common genotype was HPV 52 (23.3%) followed by HPV 16 (13.5%) and HPV 58 (12.7%). Of the 179 cases (26.2%) with low-risk HPV infection, the most common HPV genotype was HPV 61 (25.7%), followed by HPV 81 (21.2%) and HPV 55 (15.6%) (Fig. 3).

## Discussion

There are regional differences in cervical HPV infection.^8^ The prevalence of infection with any HPV was 38.4% and prevalence of infection with disease-associated HPV was 19.9% in females in the United States in 2018.^9^ However, the infection rate of female HPV high-risk screening in Putian, China, was 9.6% and mainly involved single infections; In addition, HPV 16, HPV 52, and HPV 58 were closely related to the severity of cervical cytology.^10^ Understanding the epidemiological characteristics of HPV infection in this area is very important for the prevention and treatment of cervical cancer. In this study, HPV testing was performed in 4746 healthy subjects, and 682 cases of HPV infection were identified, yielding a 14.4% infection rate. High-risk HPV infection is the main cause of cervical cancer.^11^

High-risk HPV-DNA is often integrated into the host cell DNA. The integration of HPV-DNA mutates or deletes E6 and E7 oncoprotein integration genes and leads to inactivation and loss of function of the E2 protein encoded by the E2 gene, resulting E6 and E7 gene overexpression, enhanced transformation ability, and cell transformation or carcinogenesis. This study showed that among the 682 cases of HPV infection, high-risk HPV infection accounted for 503 cases (73.8%), and the most common genotype was HPV 52 (23.3%), followed by HPV 16 (13.5%) and HPV 58 (12.7%). Of the 179 cases (26.2%) with low-risk HPV infection, the most common genotype was HPV 61 (25.7%), followed by HPV 81 (21.2%) and HPV 55 (15.6%).

Yang et al.^12^ found that the highest infection rates of high-risk HPV were HPV 16, 52, and 58, whereas the highest infection rates of low-risk types were HPV 6, 11, and 43. The results of this study can provide a reference for the prevention and treatment of cervical cancer in Jinan.

The difference in the infection rate in different regions may be related to the diversity of HPV detection methods and the imbalance in infection factors. According to previous reports, the age-specific HPV infection rate tends to have a bimodal distribution in China and worldwide. Specifically, young women (<25 years old) and old women (after 60 years old) exhibit the highest levels of infection.^13^ However, HPV infection in young women is often a transient infection, so cervical HPV testing is recommended for women aged 30 years and older. Most individuals undergoing physical examination are working, so there are fewer individuals younger than 25 years of age and older than 60 years. This study found that the age group with the highest HPV infection rate was 30–39 years old, accounting for 35.6%, and the proportion of individuals with single infection was the highest in this age group.

Regarding mixed infections, the rate was the highest in the 30- to 39-year-old group, accounting for 19.8% followed by the 50- to 59-year-old group, accounting for 19.3%. In addition, the infection rate was higher in people aged 31–50 years, which was consistent with the high incidence of cervical cancer in women between 30 and 55 years old. Worldwide, HPV 16, HPV 18, HPV 31, HPV 58, and HPV 52 are the most common subtypes of infection, and HPV 16/18 infection is the most common. However in Asia, the most common subtype is HPV 52. This point has also been verified in this study. High-risk HPV 52 is the most common strain in this area followed by HPV 16 and HPV 58, which is consistent with previous survey results in China.^14^ The infection rate is the highest in the <21-year-old group and 51- to 60-year-old group.^15^

The top 3 infection rates in the Hebei area were HPV 16, HPV 58, and HPV 52. The 15- to 25-year-old and ≥56-year-old groups had higher HPV infection rates.^16^ The difference in the distribution of high-risk HPV types may also be related to the different detection methods used. These results suggest that HPV 52 and 58 infections should also be of concern.

In this study, the 30- to 39-year-old group had the highest levels of mixed infections followed by the 50- to 59-year-old group. This finding may be explained by the fact that individuals in the 30- to 39-year-old groups are more sexually active, which is a factor associated with HPV infection. The average age of Chinese women entering perimenopause is 46 years old, and the average age of menopause is 48–52 years old.^17^ HPV infection can easily occur at the second peak of HPV prevalence in older women may be attributed to the persistent HPV infection or reactivation of a latent HPV infection, which was associated with physiological and immunological disorders resulting from hormone fluctuations during their menopausal transition.^18^

Through this investigation, our research group found that the vast majority of people who underwent physical examination were tested for HPV for the first time, indicating that cervical cancer screening programs have not been implemented regularly in Jinan. Tota et al.^19^ analyzed two large prospective randomized double-blind controlled trials (bivalent HPV vaccine group (*n* = 10,750) and nonvaccine control group (*n* = 10,846). Among individuals receiving the bivalent HPV vaccine, the vaccine not only had a protective effect on HPV 16/18 infection but also had a cross-protective effect on HPV 35, HPV 52, HPV 58, and HPV 68. The cross-immunization of HPV affects not only the infection spectrum of HPV but also the efficacy of the vaccine. Studies have shown that among patients positive for HPV 16 and HPV 18, the risk of contracting other high-risk HPV strains was significantly lower than that of HPV 16 and HPV 18 negative individuals.^20^

With the introduction and popularization of prophylactic HPV vaccines in China, one aspect we should pay attention to is whether there is type substitution in the population of HPV vaccines. Specifically, the decrease in infections by the HPV strain targeted by the vaccine leads to an increase in the HPV infection rate of other strains not targeted by the vaccine, which weakens the preventive and therapeutic effect of vaccines as a whole.

Finally, the prevalent HPV strains in Jinan City differ from those in other regions. This study also has limitations that deserve attention. First, this is a single-center study, and selection bias in the study limited the extension of the results from this study to other populations. Second, we did not collect the demographic data, such as vaccination status, history of prior HPV infection, hysterectomy status, and tobacco use and so on. Third, there was a lack of pathological or cytological analyses of the participants' cervix, which provide important prognostic information, as seen in some reports. At last, a follow-up study should be done to track changes in genotype, as there is a close relationship between cervical carcinoma and long-term persistent high-risk HPV infections.

## Conclusion

In short, the study shows that the infection rate of HPV in healthy female patients is relatively high, and the mixed infection rate is as high as 24.6%, which is clinically important. To further standardize cervical cancer screening programs and develop new HPV vaccines, we should further study and formulate different cervical cancer prevention and treatment strategies based on regional differences.

## Data Sharing Statement

All patient data were stored in the electronic database YIDUCLOUD (http://192.168.160.2). The dataset that supports the findings is not publicly available because it contains information that could compromise research participant privacy. Limited deidentified datasets may be available from the authors upon reasonable request.

## Abbreviations Used

HPV: human papillomavirus
HR-HPV: high-risk human papillomavirus strains
LR-HPV: low-risk human papillomavirus strains
PCR: polymerase chain reaction
